# Anti-plasmodial activity of proteasome inhibitors against various *Plasmodium* species: a preclinical evaluation

**DOI:** 10.1128/aac.00382-26

**Published:** 2026-09-30

**Authors:** Nguyen Van Truong, Tuyet-Kha Nguyen, Nguyen Sy Thau, Thi-Thanh Hang Chu, Jin-Hee Han, Sung-Hun Na, Won-Sun Park, Wan-Joo Chun, Joo Hwan No, Feng Lu, Robert W. Moon, Eun-Taek Han

**Affiliations:** 1 Department of Tropical Medicine, Kangwon National University School of Medicine 85082, Chuncheon-si, Gangwon-do, Republic of Korea; 2 Department of Infectious Diseases, 103 Military Hospital 126754, Hanoi, Vietnam; 3 Institute of Clinical Infectious Diseases, 108 Military Central Hospital, Hanoi, Vietnam; 4 Department of Obstetrics and Gynecology, Kangwon National University School of Medicine 85082, Chuncheon, Gangwon-do, Republic of Korea; 5 Department of Physiology, School of Medicine, Kangwon National University 34962, Chuncheon, Gangwon-do, Republic of Korea; 6 Department of Pharmacology, School of Medicine, Kangwon National University 34962, Chuncheon, Gangwon-do, Republic of Korea; 7 Chemical and Structural Biology of Pathogens, Discovery Biology, Institut Pasteur Korea 201612, Seongnam-si, Gyeonggi-do, Republic of Korea; 8 School of Basic Medical Sciences & School of Public Health, Faculty of Medicine, Yangzhou University, Yangzhou, China; 9 Department of Infection Biology, Faculty of Infectious and Tropical Disease, London School of Hygiene and Tropical Medicine 4906, London, United Kingdom; The Children's Hospital of Philadelphia, Philadelphia, Pennsylvania, USA

**Keywords:** proteasome inhibitor, *Plasmodium*, antimalarial drug, ONX-0914

## Abstract

The discovery of novel anti-malarial drugs has become urgent due to increasing drug resistance. Our previous work identified ONX-0914, which targets parasite proteolytic systems, as promising against *Plasmodium falciparum*. This study aims to further evaluate the antiplasmodial activity of ONX-0914 and related proteasome inhibitors (PIs) against various *Plasmodium* strains. The *in vitro* antimalarial activity of the inhibitors was evaluated against the *Plasmodium knowlesi* A1H1 strain and compared with that against the chloroquine (CQ)-sensitive *P. falciparum* 3D7 strain. Potent synergistic interactions between compounds and artemisinin (ART) were analyzed against PkA1H1 and ART-resistant PfDd2R539T(+) using an isobologram. To explore the mode of action, the disruption of the ubiquitin-proteasome system (UPS) in PkA1H1 and Pf3D7 was investigated. *In vivo* efficacy of ONX-0914 was further validated against CQ-resistant *Plasmodium yoelii*. PIs significantly inhibited PkA1H1 proliferation with low-nanomolar IC_50_ values; ONX-0914 and LU-005i were more potent against PkA1H1 than Pf3D7, and effectively blocked multiple blood stages. Inhibition of parasite growth correlated with potent disruption of UPS at nanomolar concentrations (IC_50_ < 50 nM). ONX-0914 demonstrated strong synergistic activity with ART and other PIs (∑FIC50 < 0.8). *In vivo*, ONX-0914 significantly suppressed *P. yoelii* infection, improving lifespan, with ⁓84% efficacy at an intraperitoneal (IP) dose of 20 mg/kg, and ⁓90% in combination with artemisinin at a dose of 10 mg/kg (IP). Further optimization of the structure and dosing regimen is able to enhance the therapeutic potential of PIs as potential antimalarial drug candidates.

## INTRODUCTION

Malaria continues to pose a global health burden, with approximately 263 million cases and 597,000 deaths reported in 2023 (1). The growing prevalence of antimalarial drug resistance, especially in Africa, the Americas, and Southeast Asia, has further intensified this situation (1). Artemisinin (ART)-based combination therapies (ACTs) remain the standard treatment for uncomplicated *Plasmodium falciparum* malaria; however, recent therapeutic efficacy studies have revealed rising treatment failures. These failures are mainly attributed to mutations in the partner drug’s genotype that reduce its effectiveness (2–5). Therefore, it is critical to expand antimalarial drug discovery efforts, particularly those targeting compounds with novel mechanisms of action.

Human malaria is caused by several *Plasmodium* species, including *P. falciparum*, *Plasmodium vivax*, *Plasmodium malariae*, *Plasmodium ovale*, and *Plasmodium knowlesi*, each with distinct biological characteristics and drug-susceptibility profiles (6). *P. falciparum* is a major worldwide species with the most severe cases observed and has developed widespread resistance to multiple antimalarials, such as chloroquine (CQ) and ART (1). In contrast, stable long-term *in vitro* maintenance of *P. vivax* remains unsuccessful despite considerable effort and has emerged with resistance to chloroquine and primaquine, particularly in Southeast Asia and Oceania (1, 7, 8). Meanwhile, *P. knowlesi*, originally known as a zoonotic malaria parasite, has emerged as a crucial cause of human malaria in Southeast Asia and can cause severe and potentially fatal disease. In addition to its clinical relevance, *P. knowlesi* has been adapted to continuous *in vitro* culture with human erythrocytes, providing a valuable experimental platform for comparative antimalarial studies and for evaluating species-specific drug responses (9–11). Evaluation of species-specific drug responses is necessary for antimalarial drug development, and a broad-spectrum antimalarial agent effective against multiple species is required.

The ubiquitin-proteasome system (UPS) plays a vital role in eukaryotic cells by degrading misfolded or damaged proteins and has become a key target for therapeutic development. In *Plasmodium*, the UPS is indispensable for parasite growth, replication, and stress adaptation (12). Unlike many established antimalarial drugs that primarily target hemoglobin digestion, heme detoxification, or parasite metabolism, proteasome inhibitors (PIs) disrupt protein homeostasis through a distinct mechanism of action. This mechanistic difference offers a strong rationale for their development as novel antiplasmodial agents and for their evaluation in combination with existing antimalarial drugs. Furthermore, the UPS is highly conserved across *Plasmodium* species, with substantial orthology of its catalytic subunits and protein homeostasis machinery (13–15). Because continuous long-term *in vitro* culture of *P. vivax* remains challenging, comparative evaluation in complementary *Plasmodium* models represents an important strategy for antimalarial drug discovery. Consequently, assessment of proteasome inhibitors in *P. falciparum*, *P. knowlesi*, and rodent malaria models may offer insight into whether inhibition of this conserved essential pathway can be broadly applied to human malaria parasites, including *P. vivax* (16–18). Indeed, several PIs, such as MG132 and TDI 8304, have exhibited potent activity against drug-resistant *P. falciparum* strains (19–22). Nonetheless, obstacles such as poor bioavailability, limited *in vivo* efficacy, and toxicity have hindered the clinical translation of PIs (20, 23–25).

In our previous work, we identified ONX-0914, a selective proteasome inhibitor, which demonstrated potent antiplasmodial activity against *P. falciparum*, synergistic interaction with ART *in vitro*, and significant potency in a chloroquine-sensitive *Plasmodium berghei* mouse model (22). These findings established proof of concept that selective proteasome inhibition is a promising antimalarial strategy. However, the extent to which this activity is conserved across other clinically relevant *Plasmodium* species, retained in drug-resistant parasite models, and enhanced when combined with other proteasome inhibitors remains unknown. These attributes—potent activity against multiple *Plasmodium* species, efficacy against resistant parasites, and synergistic interaction with existing antimalarial drugs—are consistent with the Medicines for Malaria Venture (MMV) target candidate profiles (26). However, extensive analysis of ONX-0914 across multiple *Plasmodium* species, including *P. knowlesi* and an ART-resistant *P. falciparum* strain, as well as validation in CQ-resistant mouse models and detailed assessment of synergy with other PIs *in vitro* and combination with ART *in vivo*, remains incomplete.

Because antimalarial susceptibility and resistance phenotypes may vary among *Plasmodium* species, evaluating candidate compounds across complementary parasite models may provide a more comprehensive assessment than relying on a single species. Accordingly, *P. falciparum*, *P. knowlesi*, and *Plasmodium yoelii* were selected to address distinct but complementary questions regarding antiplasmodial activity, mechanism of action, combination efficacy, and *in vivo* proof-of-concept activity. Therefore, this study aims to extend and strengthen previous findings through an integrated investigation. Specifically, we evaluated *in vitro* activity against *P. knowlesi* relative to *P. falciparum*, assessed synergistic interactions with other PIs against various strains, examined disruption of the ubiquitin-proteasome system, and validated *in vivo* efficacy in monotherapy and in combination with ART using a *P. yoelii* CQ-resistant mouse malaria model. By incorporating complementary *Plasmodium* species, mechanistic assays, and efficacy studies, we sought to determine whether PIs exhibit consistent antiplasmodial activity and favorable combination profiles across multiple experimental systems. These thorough investigations present critical insights into the potential translation of ONX-0914 and support the repositioning of PIs as promising antimalarial drug candidates.

## MATERIALS AND METHODS

### Animals

Female BALB/c mice (6–8 weeks old; 25 ± 2 g) were purchased from Orient Bio (Republic of Korea). Mice were housed at controlled conditions (25°C ± 2°C; 12 h light/dark cycle) with laboratory diet and water *ad libitum* and acclimatized for 4 days before the experiment.

### Compounds

ONX-0914, epoxomicin (EPO), LU-005i, bortezomib (ChemScene), atovaquone (ATQ) (Sigma‒Aldrich), and artemisinin (ART) (Merck) were prepared in 100% dimethyl sulfoxide (DMSO; Sigma‒Aldrich; 10 mM stock). Chloroquine diphosphate salt (CQ; Sigma‒Aldrich) was dissolved in distilled water (DDW), stored at −20°C, and diluted as needed.

### Parasite strains and mammalian cells

*P. falciparum* (3D7, NF54luc-GFP, and Dd2R539T(+)), *P. knowlesi* A1H1, and *P. yoelii* XL strains were obtained from BEI Resources (27). HEK293T cells were purchased from American Type Culture Collection (28). Human erythrocytes were obtained from Kangwon National Hospital.

### Toxicity to a human cell line

HEK293T cells were used for cytotoxicity assays using Cell Counting Kit-8 (CCK-8, Dojindo), as described previously (29, 30). A total of 90 µL of HEK293T cells (5,000 cells/well) was seeded into a 96-well plate and exposed to 10 µL of serially diluted test inhibitors for 48 h. Cell viability was assessed with the CCK-8 reagent, and absorbance (Abs) at 450 nm was measured with a SpectraMax M5 plate reader (Molecular Devices). The half-maximal cytotoxic concentration (CC_50_) was derived from log-linear interpolation of the cytotoxicity curves (31).

### *In vitro* culture of *Plasmodium* parasites

*P. falciparum* blood-stage parasites were cultured in O^+^ human red blood cells (RBCs) in RPMI 1640 media (Sigma‒Aldrich) supplemented with 100 µM hypoxanthine, 12.5 µg/mL gentamicin, 0.5% (wt/vol) Albumax I (Thermo Fisher), and 2 g/L sodium bicarbonate (WELGENE Inc.) at 37°C in N_2_ containing 1% O_2_ and 5% CO_2_, as described previously (32). *P. knowlesi* parasites were maintained in modified medium RPMI 1640 containing 100 µM hypoxanthine, 12.5 µg/mL gentamicin, 2 g/L sodium bicarbonate, 10 mM D-glucose, 2 mM L-glutamine, 0.5% (wt/vol) Albumax II (Thermo Fisher), and 10% (vol/vol) horse serum (Thermo Fisher) (33).

### Growth inhibition assay

The *in vitro* growth inhibition assay was conducted as previously studied (34, 35). *P. falciparum* culture was double-synchronized using 5% (wt/vol) sorbitol to obtain ring-stage parasites (36). For *P. knowlesi*, schizonts were enriched using a 40%/70% Percoll gradient, then cultured for an additional 4–8 h to generate the ring stage (37). Ring-stage *P. falciparum* and *P. knowlesi* parasites were maintained under identical conditions in 96-well plates, with each well containing 100 µL of culture, adjusted to 2% hematocrit and 1% parasitemia. Serial dilutions of compounds, dissolved in DMSO (0.1%), were added to the treatment wells. Untreated parasite cultures served as control wells, and wells containing uninfected RBCs were used to exclude background. Cultures were incubated at 37°C in a humidified culture chamber for 1.5 cycles (72 h for *P. falciparum* and 42 h for *P. knowlesi*).

Parasitemia was quantified by SYBR Green I (0.2 × dilution, Thermo Fisher) staining and analyzed on an Accuri C6 flow cytometer (Accuri Cytometers, Inc.). Growth inhibition was calculated using the formula: % growth inhibition = 100 − 100 × ([intervention well parasitemia − uninfected well parasitemia] / [infected well parasitemia − uninfected well parasitemia]). The half-maximal inhibitory concentration (IC_50_) value was determined using the equation (log[inhibitor] vs response‒variable slope [four parameters]) (31).

### Drug combination studies

The fixed-ratio isobologram method was used to study *in vitro* interactions between compounds against PfDd2R539T(+) and PkA1H1, as described in previous studies (21, 22). The combinations of ATQ vs ART, EPO vs ART, and ONX-0914 vs ART against PfDd2R539T(+) strain were included as reference controls based on previously reported drug interactions (22, 38, 39). Specifically, the ATQ vs ART served as a reference for antagonistic interactions, whereas the EPO vs ART and ONX-0914 vs ART served as references for synergistic interactions. Drug mixture sets (ATQ vs ART, EPO vs ART, ONX-0914 vs ART, ONX-0914 vs EPO, ONX-0914 vs LU-005i, and ONX-0914 vs bortezomib) with set ratios of 1:0, 4:1, 3:2, 2:3, 1:4, 0:1 were serially diluted 10-fold to generate solutions with seven concentrations. The mixture sets were dispensed in duplicate into 96-well microplates and incubated with PfDd2R539T (+) and PkA1H1 ring-stage parasites for 1.5 cycles. The fractional inhibitory concentration (FIC) was determined by dividing the IC_50_ of each drug in combination by the IC_50_ when used alone. Synergy was defined as∑FIC50 < 0.8, whereas antagonism was indicated by ∑FIC50> 1.5.

### Kill rate assay

In the kill rate assay, relative bioluminescence intensities were measured using PfNF54luc-GFP to enable quantitative luminescence-based kinetic measurements as described in previous studies (21). A comparable luciferase-expressing PkA1H1 line was not available; therefore, kill-rate analysis was not performed in PkA1H1. Briefly, parasite viability was assessed after 6 h of exposure to serial dilutions of each compound by measuring bioluminescence.

### Effects of inhibitors on parasite morphology and stage-specific development

The procedure used for evaluating the effects on morphology and stage-specific development of PkA1H1 was adapted from a previous study (21). Briefly, ring-stage parasites were treated with DMSO (0.1%) or inhibitors (⁓3 × IC_50_ value) and incubated for 28 h. Parasite morphology was evaluated at various time points (0 h, 6 h, 12 h, 24 h, and 28 h) on Giemsa-stained thin smears, scoring at least 1,000 RBCs. To determine stage-specific inhibition, after tight synchronization at the ring (R) stage, the parasites were cultured for 12 h to obtain early trophozoites (T) or for 24 h to reach schizonts (S). The compounds were applied following the same protocol used for ring-stage growth inhibition. IC_50_ values were calculated for each treatment stage and compared.

### Effect on ubiquitinated proteins

The assay was conducted as described in a previous study with minor modifications (19). To assess UPS inhibition, 25 mL cultures of early trophozoite-stage Pf3D7 and PkA1H1 parasites (at 11% parasitaemia; 2% hematocrit) were treated with DMSO (0.1% final), inhibitors (conc. 1 µM), or left untreated; the latter served as controls. After 10 h of incubation, cultures were centrifuged, and parasite pellets were subsequently lysed with 0.01% saponin for 10 min, followed by PBS washes. Pellets were denatured in 150 µL of reducing SDS‒PAGE sample buffer, boiled for 10 min, and then centrifuged at 27,216 × *g* for 15 min at room temperature (RT). Supernatants were separated on 9% SDS‒PAGE gels and then transferred to an Immobilon-P membrane (Millipore). Membranes were blocked with 5% skim milk at RT for 1 h and probed with mouse anti-ubiquitin antibodies (dilution 1/500 in blocking buffer; Santa Cruz Biotechnology) or mouse anti-GAPDH (dilution 1/5,000 in blocking buffer; Sigma‒Aldrich), followed by goat anti-mouse IRDye (dilution 1/10,000 in blocking buffer; Invitrogen). Signals were visualized using an Odyssey infrared imaging system (LI-COR Bioscience), quantified by densitometry using software (Image J2), and the ubiquitin signal was normalized to GAPDH.

For immunoblot analysis, equal aliquots of each protein lysate from the same biological samples were loaded onto two parallel SDS-PAGE gels and conducted simultaneously under identical electrophoresis, transfer, blocking, antibody incubation, and imaging conditions. One membrane was probed with the anti-ubiquitin antibody, whereas the second membrane was probed with the anti-GAPDH antibody as a loading control. During figure preparation, corresponding lanes from the two membranes were assembled into composite figures to align ubiquitin and GAPDH signals from the same samples. Non-adjacent lane junctions are indicated by thin black dividing lines in the figures.

### Analysis of proteasome activity via the proteasome-GLO system

Proteasome activity was assessed using a modified version of a previous study (16). Trophozoite-stage Pf3D7 and PkA1H1 cultures (3% hematocrit and 4% parasitemia) were exposed to varying concentrations of the inhibitors. After one replication cycle, 0.05% (wt/vol) saponin was added to selectively release RBC cytoplasmic components. Parasite pellets were washed three times with ice-cold PBS and lysed in ice-cold water (pH 4.5). Following centrifugation at 760 × *g* for 10 min, 1 µg of total supernatant protein was mixed with an equal volume of Proteasome-GLO (chymotrypsin-like) assay reagent (Promega). Luminance was recorded every minute for 3 h using a BioTek Synergy H1 plate reader. Proteasome activity was quantified from readings between 60 and 90 min. Values were normalized to the 0.1% DMSO control (100% proteasome-GLO signal). IC_50_ values were calculated using the equation (log[inhibitor] vs response—variable slope [four parameters]) (31).

### Antiplasmodial activity assay in a mouse model

*In vivo* efficacy was evaluated in *P. yoelii* XL-infected BALB/c mice, following the previously described protocol with modifications (40). Female BALB/c mice (5 mice in each group) were inoculated intraperitoneally with 10^6^ *P. yoelii* XL-infected RBCs obtained from passage mice, except for the negative control group. ART and ONX-0914 were dissolved in the vehicle (10% DMSO, 3% ethanol, 7% Tween-80, and 80% DDW) (VZV), vortexed thoroughly, and administered once daily at 6, 24, 48, and 72 h post-infection. Single-agent treatments consisted of 20 mg/kg (intraperitoneal [IP]), while combination therapy included 10 mg/kg of each compound (IP) at a volume of 200 µL. Control groups received vehicle (200 µL), 10 mg/kg CQ dissolved in DDW, or no treatment (positive controls) or no infection (negative control). Parasitemia was determined by counting 1,000 erythrocytes on Giemsa-stained tail-vein blood smears and observation under a 100 × objective. Twenty-four hours after the final dose (4 days), the percent suppression was calculated using the formula: % 4-day suppression = 100 – 100 × parasitemia (intervention)/parasitemia (positive). Additional parameters, including adverse events, mean survival days (MSDs), survival rates (SRs), clinical signs, and body weights, were recorded. Mice treated with ONX-0914, ART, or combination therapy were monitored until 32 days post-infection (DPI) to evaluate recrudescence.

### Histopathology

Organs (brain, heart, lung, liver, and kidney) were collected post-mortem from at least three mice per group, selected for having the highest parasitaemia. Mice were monitored daily, and those that died during infection or reached the humane endpoint (2 weeks) were necropsied. Tissues were rinsed in PBS, fixed in 10% buffered formalin, and assessed for changes in color and size (41). Organ size was calculated as the height × width (mm). For histopathology, organs were cut at an appropriate thickness and embedded in paraffin. The paraffin-embedded tissues were sectioned at 3 μm thickness and stained with hematoxylin and eosin. Slides were examined under a light microscope (BX53, Olympus), and organ lesions were scored as minimal (1+), mild (2+), moderate (3+), or severe (4+).

### Statistical analysis

The data were analyzed using GraphPad Prism (GraphPad Software) and Microsoft Excel 2018 (Microsoft). To compare experimentally measured values across groups, Student’s *t*-tests and Mann–Whitney *U-*tests were used. *P* < 0.05 was considered to show a significant difference.

## RESULTS

### *In vitro* cytotoxicity and inhibitory effects of proteasome inhibitors on *Plasmodium* strains

Cytotoxicity profiling in HEK293T cells demonstrated that all PIs had CC_50_ values greater than 100 µM, comparable to those of CQ and ART, indicating low toxicity toward human cells (Table 1). Across *Plasmodium* strains, the PkA1H1 strain showed greater sensitivity to the reference drugs CQ and ART than the Pf3D7 strain. Among these PIs, epoxomicin, ONX-0914, and LU-005i exhibited potent antiplasmodial activity against both PkA1H1 and Pf3D7 strains, with IC_50_ values in the low nanomolar range. Notably, ONX-0914 and LU-005i demonstrated significantly stronger inhibition of PkA1H1 than Pf3D7 (*P* < 0.05), consistent with CQ and ART. In contrast, Bortezomib demonstrated low inhibition against both strains (Table 1). The selectivity index (SI) (HEK293T CC_50_/*Plasmodium* IC_50_) exceeded 5,000 for epoxomicin, ONX-0914, and LU-005i across both Pf3D7 and PkA1H1, similar to CQ and ART, highlighting strong parasite selectivity for these inhibitors. In contrast, bortezomib showed lower selectivity than other inhibitors (Table 1).

**TABLE 1:** Summary of *in vitro* cytotoxicity (CC_50_) against HEK293T and antimalarial activity of each compound (IC_50_) against *Plasmodium^c^*

Compound	IC_50_, nM ring stage		CC_50_, µM	Selective index (CC_50_/IC_50_)^*a*^	*P* value^*b*^
	Pf3D7	PkA1H1			
CQ	20.1 ± 1.4	4.3 ± 0.1	180.2 ± 51.9	8,965.2	0.0039
ART	19.3 ± 1.0	1.1 ± 0.5	>100	>5,189.4	0.0004
Epoxomicin	0.1 ± 0.0	2.5 ± 0.4	683.1 ± 234.1	6,831,000	0.0136
ONX-0914	22.0 ± 3.7	8.4 ± 0.1	232.0 ± 93.6	10,545.5	0.0351
LU-005i	67.5 ± 4.7	41.9 ± 8.6	881.5 ± 32.7	13,059.3	0.0008
Bortezomib	>200	>200	194.1 ± 142.4	<970.5	–

^*a*^ The selective index was calculated as the ratio of CC50 per IC50 against 3D7.

^*b*^ *P* value was determined by pair comparison of IC50 against 3D7 and A1H1 using a two-tailed Student’s *t*-test; –, not determined.

^*c*^ IC50 and CC50 values represent the mean ± SD from three independent assays, each performed in duplicate, collected per experiment. IC50 and CC50 were determined by fitting to the Log vs response-variable slope equation in GraphPad Prism.

### Effects of proteasome inhibitors on parasites in the erythrocytic stage

**Fig 1:**
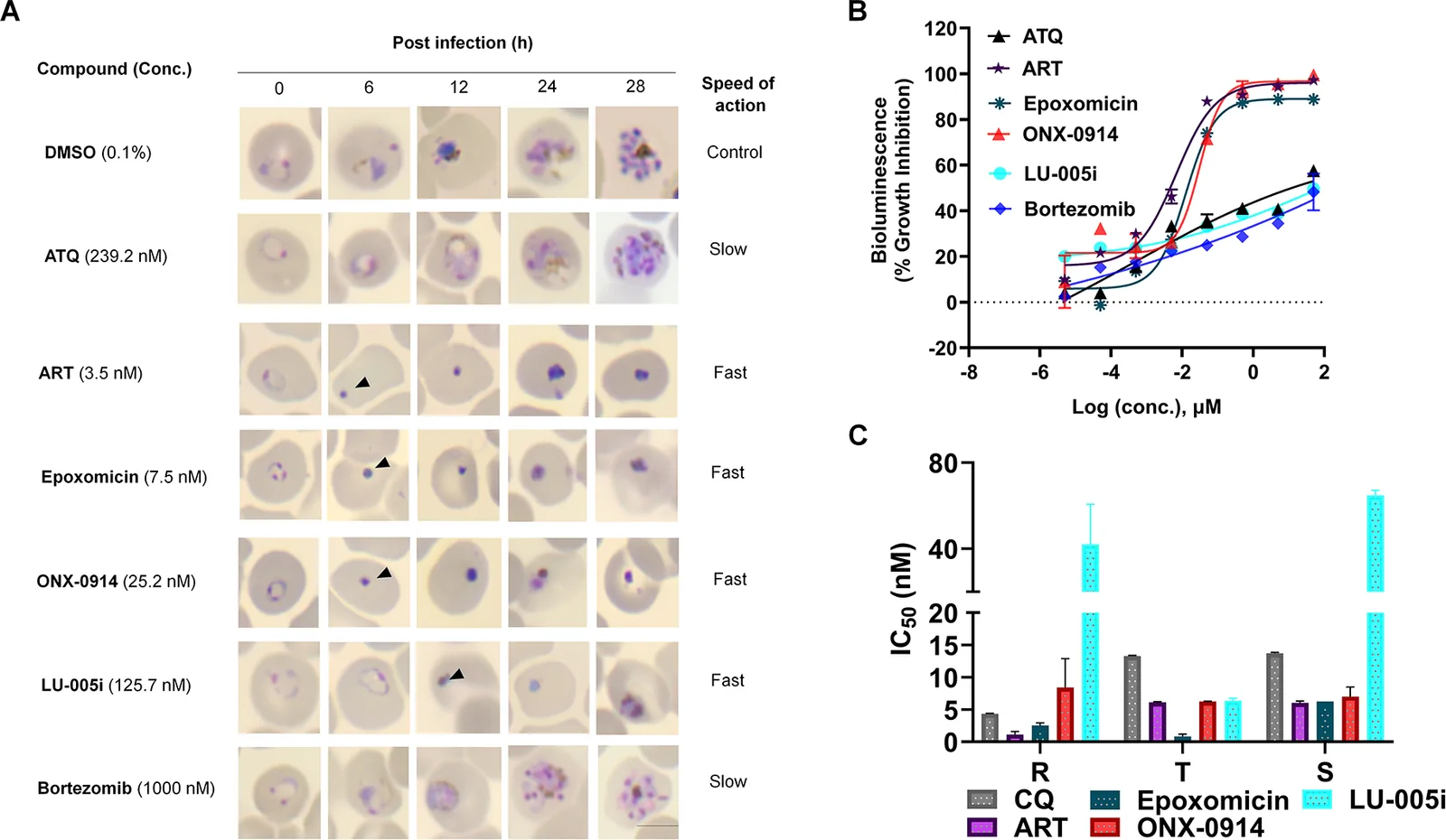
Morphological effects, kill rates, and stage-specific activity of proteasome inhibitors. (**A**) Ring-stage *P. knowlesi* A1H1 parasites were cultured with 0.1% DMSO or the indicated inhibitors (at 3 × IC_50_ value) for 28 h, after which they showed morphological disruption. ART, epoxomicin, and ONX-0914 induced pyknotic forms within 6 h; LU-005i produced effects after 12 h. The black arrows indicated pyknotic vacuoles. Scale bars = 5 µm. (**B**) Kill rate assay using PfNF54luc-GFP parasites after 6 h of treatment. ART (fast-kill) and ATQ (slow-kill) were used as controls. (**C**) Stage-specific IC_50_ values (R, T, and S). IC_50_ of bortezomib exceeded 1,000 nM at all stages.

Treatment with EPO, ONX-0914, and ART primarily induced marked morphological alterations in PkA1H1-infected RBCs within 6 h, including pyknotic nuclei and irreversible chromatin condensation, consistent with necrotic or apoptotic death (Fig. 1A). LU-005i treatment caused similar changes but only after 12 h of exposure. A substantial proportion of parasites treated with these inhibitors was arrested at the ring stage. In contrast, bortezomib elicited no notable morphological abnormalities after one erythrocytic cycle (Fig. 1A).

Morphological assessment of PkA1H1 suggested rapid clearance following treatment of EPO and ONX-0914. Representative microscopy images of *P. knowlesi* are shown in Fig. 1A to illustrate the stage-specific morphological effects of PIs. Corresponding morphological analyses in *P. falciparum* were not repeated here because they were reported in our previous study; the present work focuses on extending these observations to *P. knowlesi*. To quantitatively evaluate parasite killing, a bioluminescence-based kill-rate assay was conducted using the PfNF54luc-GFP strain (Fig. 1B). Following 6 h of treatment, bioluminescence was quantified as a measure of parasite viability. EPO and ONX-0914 significantly reduced viability at the early ring stage, exhibiting rapid-acting profiles comparable to ART. Their activity was significantly faster than that of LU-005i, bortezomib, and ATQ, which were slow acting. Together, these complementary findings indicate that EPO and ONX-0914 are fast-acting inhibitors of *P. falciparum* erythrocytic stages, whereas LU-005i and bortezomib are slow-acting inhibitors.

To determine stage-specific susceptibility, PkA1H1 parasites were treated at different stages: ring (R), early trophozoite (T), and schizonts (S) stages, and the IC_50_ values were determined after 1.5 cycles (Fig. 1C). EPO, ONX-0914, and LU-005i disrupted PkA1H1 with low nanomolar potency (IC_50_ < 100 nM) across all stages tested (*P* > 0.05). The uniformity in IC_50_ values between R, T, and S stages likely reflects the incubation period, which enables these inhibitors to inhibit not only trophozoites and schizonts but also newly formed rings in the subsequent cycle. Together, the morphological changes, speed of clearance, and stage-specific IC_50_ values demonstrate that EPO and ONX-0914 rapidly inhibit all asexual erythrocytic stages.

### Analysis of ubiquitination and proteasome activity

**Fig 2:**
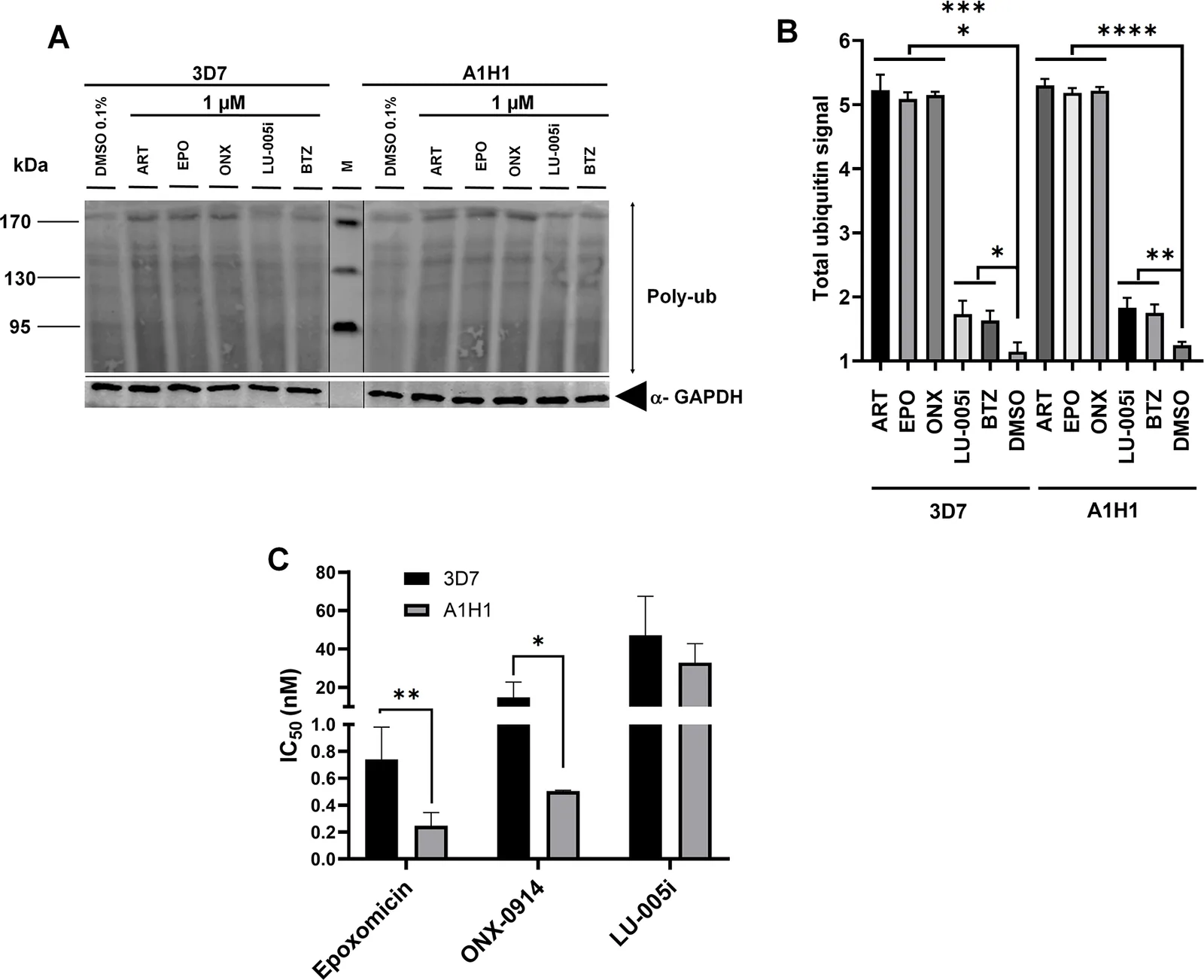
Mechanistic basis of proteasome inhibitor activity against *Plasmodium* parasites. (**A**) Western blot revealed significantly more intense high-molecular-weight ubiquitinated proteins in the ART-, epoxomicin-, and ONX-0914-treated parasites (conc. 1 µM) vs DMSO in *P. knowlesi* and *P. falciparum*. Anti-GAPDH was used as a loading control. Protein lysates from the same experiment were analyzed on two parallel SDS-PAGE gels under identical experimental conditions; one membrane was probed for ubiquitinated proteins and the other for GAPDH. Corresponding lanes were assembled for presentation, and thin black lines indicate lane junctions. (**B**) Densitometry analysis of western blots using ImageJ 2 software, and ubiquitin signal intensity was normalized to GAPDH for each lane. Normalized values confirmed significant ubiquitin accumulation. (**C**) Proteasome-GLO assay demonstrated potent *P. knowlesi* and *P. falciparum* 20S proteasome inhibition by epoxomicin, ONX-0914, and LU-005i (IC_50_ < 50 nM). ART and bortezomib had an IC_50_ value exceeding 300 nM. Data are shown as the mean ± SD (*n* = 3). Statistical significance calculated using a two-tailed *t*-test (*P < 0.01, **P < 0.001, ***P < 0.0001, and ****P < 0.00001).

To evaluate ubiquitination inhibition, parasite lysates of PkA1H1 and Pf3D7 were probed with anti-ubiquitin antibodies. Treatment with proteasome inhibitors at 1 µM induced stronger high-molecular-weight ubiquitin signals in both strains compared with the DMSO control (Fig. 2A and B). Among these inhibitors, epoxomicin and ONX-0914 produced higher ubiquitin signals than LU-005i and bortezomib, consistent with potent inhibition of the UPS. EPO, ONX-0914, and LU-005i potently inhibited Pf20S and Pk20S proteasome activity with IC_50_ < 50 nM (Fig. 2C). Notably, EPO and ONX-0914 exhibited significantly stronger inhibition of Pk20S compared with Pf20S (*P* < 0.05). Together, these findings confirm the compounds’ potent antiproteasomal activity and their contribution to suppression of asexual-stage growth across *Plasmodium* species.

### *In vitro* synergistic interaction of proteasome inhibitors

**Fig 3:**
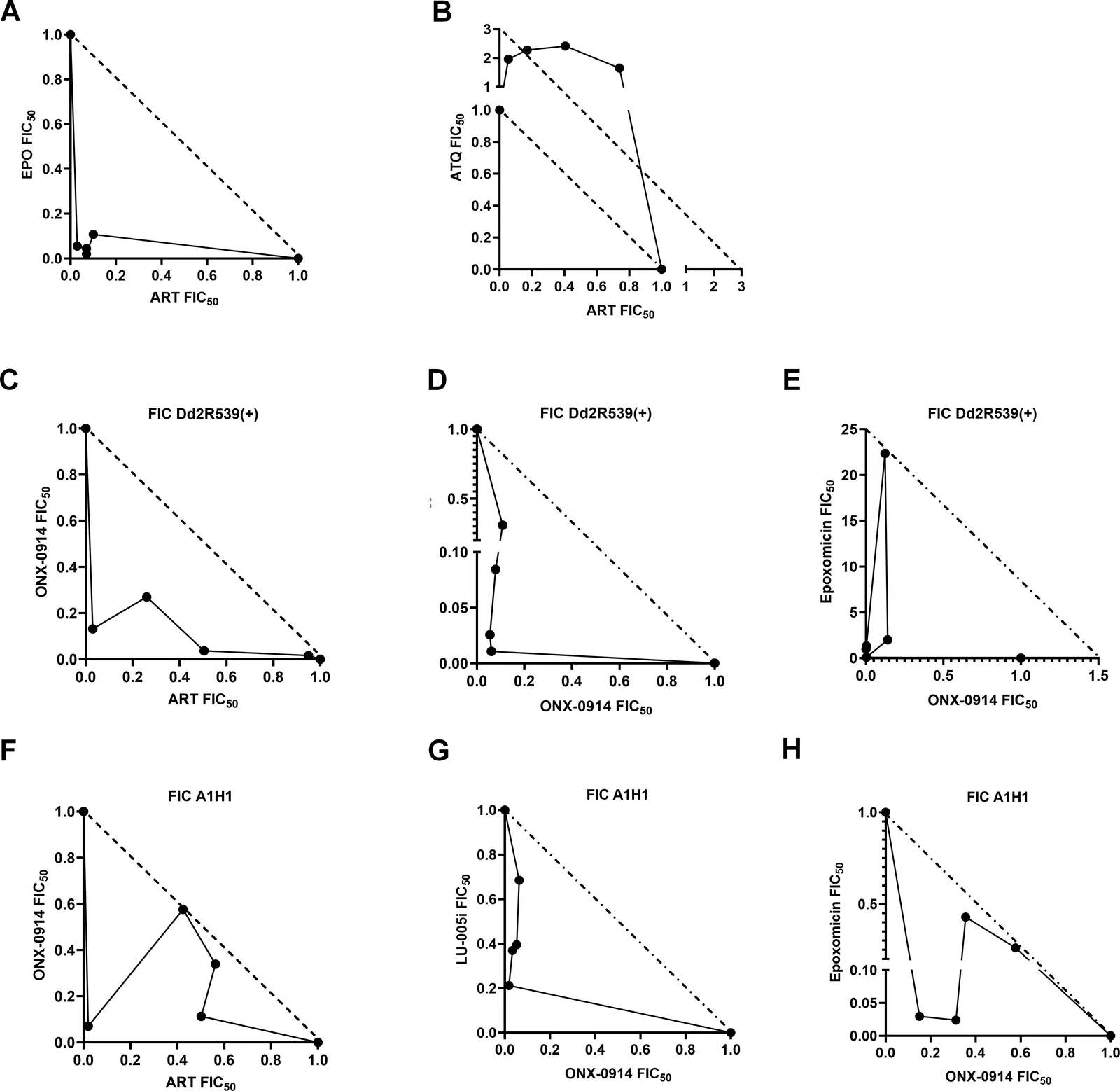
Drug interaction analyses. Fixed-ratio isobolograms against *P. falciparum* Dd2R539T(+) and *P. knowlesi* A1H1. (**A**) ART vs EPO against *P. falciparum* Dd2R539T(+), synergistic interaction. (**B**) ART vs ATQ against *P. falciparum* Dd2R539T(+), antagonistic interaction. (**C–E**) ONX-0914 vs ART, ONX-0914 vs LU-005i, and ONX-0914 vs epoxomicin against *P. falciparum* Dd2R539T(+). (**F–H**) Isobologram showing interaction between ONX-0914 vs ART, ONX-0914 vs LU-005i, and ONX-0914 vs epoxomicin against *P. knowlesi* A1H1. ONX-0914 vs ART and ONX-0914 vs LU-005i demonstrated synergism for both strains (∑FIC50 < 0.8). ONX-0914 vs epoxomicin showed synergism against *P. knowlesi* A1H1 but antagonism against *P. falciparum* Dd2R539T(+) (*n* = 3).

Isobologram analysis demonstrated potent synergistic interactions of ONX-0914 and ART against both PfDd2R539T(+) and PkA1H1 strains, with ∑FIC50 <0.8 (Fig. 3C and F). ONX-0914 also showed marked synergy with other proteasome inhibitors, including ONX-0914 vs LU-005i against both strains and ONX-0914 vs EPO against PkA1H1 ( ∑FIC50 < 0.8; Fig. 3D, G and H). In contrast, ONX-0914 exhibited an antagonistic interaction with EPO in PfDd2R539T(+) (Fig. 3E). Consistent with previous reports (22, 38, 39), the ATQ vs ART demonstrated an antagonistic interaction, whereas EPO vs ART and ONX-0914 vs ART exhibited synergistic interactions, supporting the validity of the assay conditions (Fig. 3A, B and C).

### *In vivo* antiplasmodial activity in *P. yoelii* XL-infected mouse model

**Fig 4:**
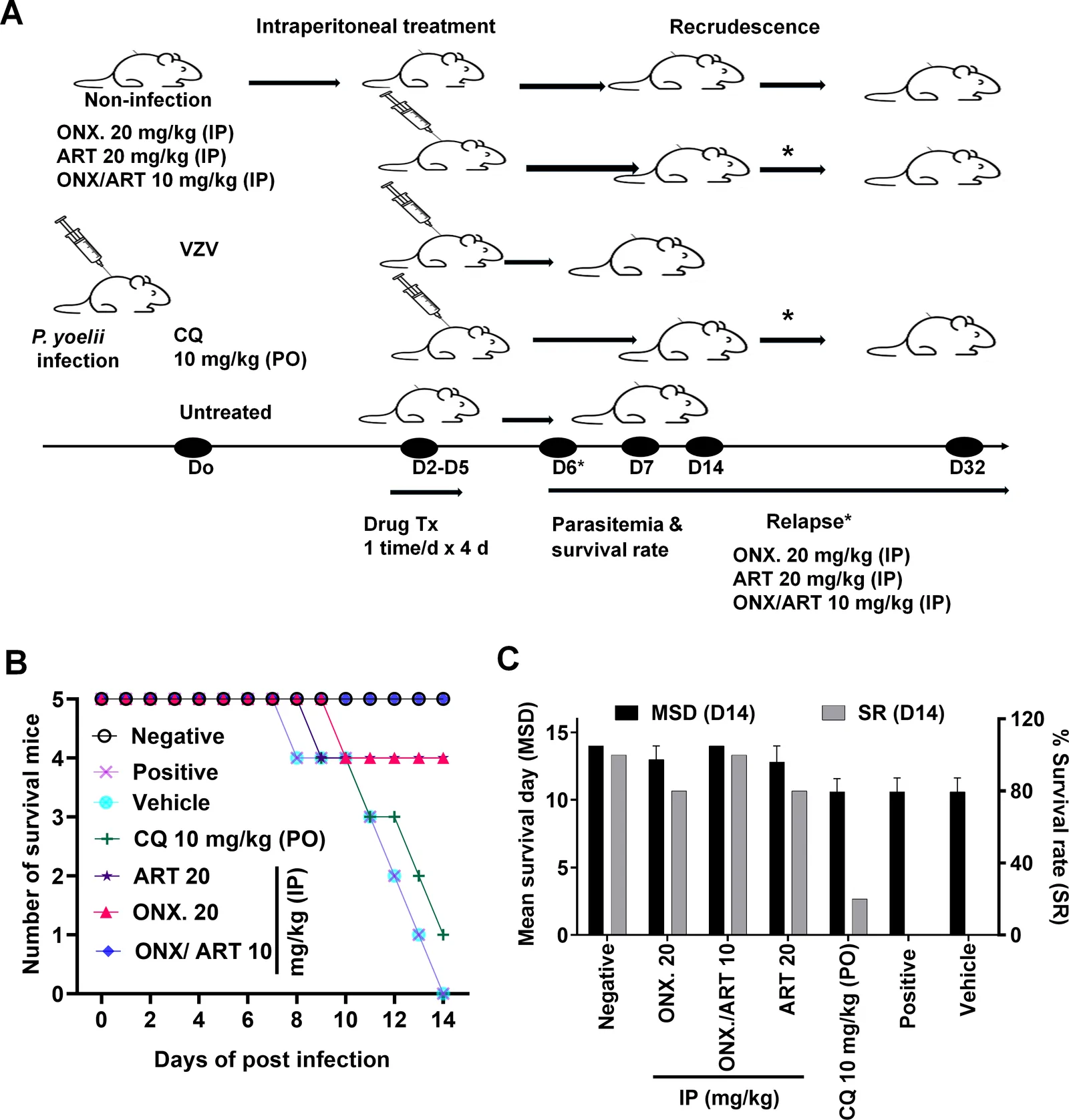
*In vivo* efficacy in *P. yoelii-*infected mouse. (**A**) Schematic overview of the *in vivo* 4-day suppression and recrudescence assays. D0, the day before treatment; Tx, drug treatment; VZV, vehicle. Mice received ONX-0914 as monotherapy (20 mg/kg, IP) or in combination with ART (10 mg/kg, IP) and were monitored for 14 days post-infection. For the recrudescence test, mice were treated with ONX-0914 (20 mg/kg, IP) alone or in combination with ART (10 mg/kg, IP) and observed for 32 days. CQ (10 mg/kg, PO) served as a drug control. (**B**) Survival of mice for each group at 15 days post-infection (DPI) and negative (uninfected mice), positive (infected mice), and vehicle groups. (**C**) SRs and MSDs for each group at 10 and 15 DPI. ONX-0914 increased the MSD and survival rate in mice, both as monotherapy and in combination. Each group included five mice.* indicates groups included in the recrudescence assay.

Among proteasome inhibitors, ONX-0914 exhibited promising antiplasmodial activity *in vitro* and was evaluated for efficacy *in vivo*, both as monotherapy and in combination with ART via IP routes in *P. yoelii-*infected mice (Fig. 4A). The antiplasmodial activity of compounds was evaluated up to 14 DPI (Fig. 4A) and recrudescence was assessed to 32 DPI (Fig. 4A). Mice treated with CQ (10 mg/kg, PO) showed a low SR (20%) and short MSD (10.6 ± 2.2 days), confirming the CQ-resistant phenotype of *P. yoelii* strain (Fig. 4B and C). In contrast, ART and ONX-0914 monotherapy at 20 mg/kg (IP) significantly improved outcomes, yielding an SR of 80% and MSD exceeding 12.8 ± 2.6 days (Fig. 4B and C). Strikingly, the ONX/ART combination at half dose (10 mg/kg each) further enhanced therapeutic efficacy, with no deaths observed, compared with either monotherapy.

**Fig 5:**
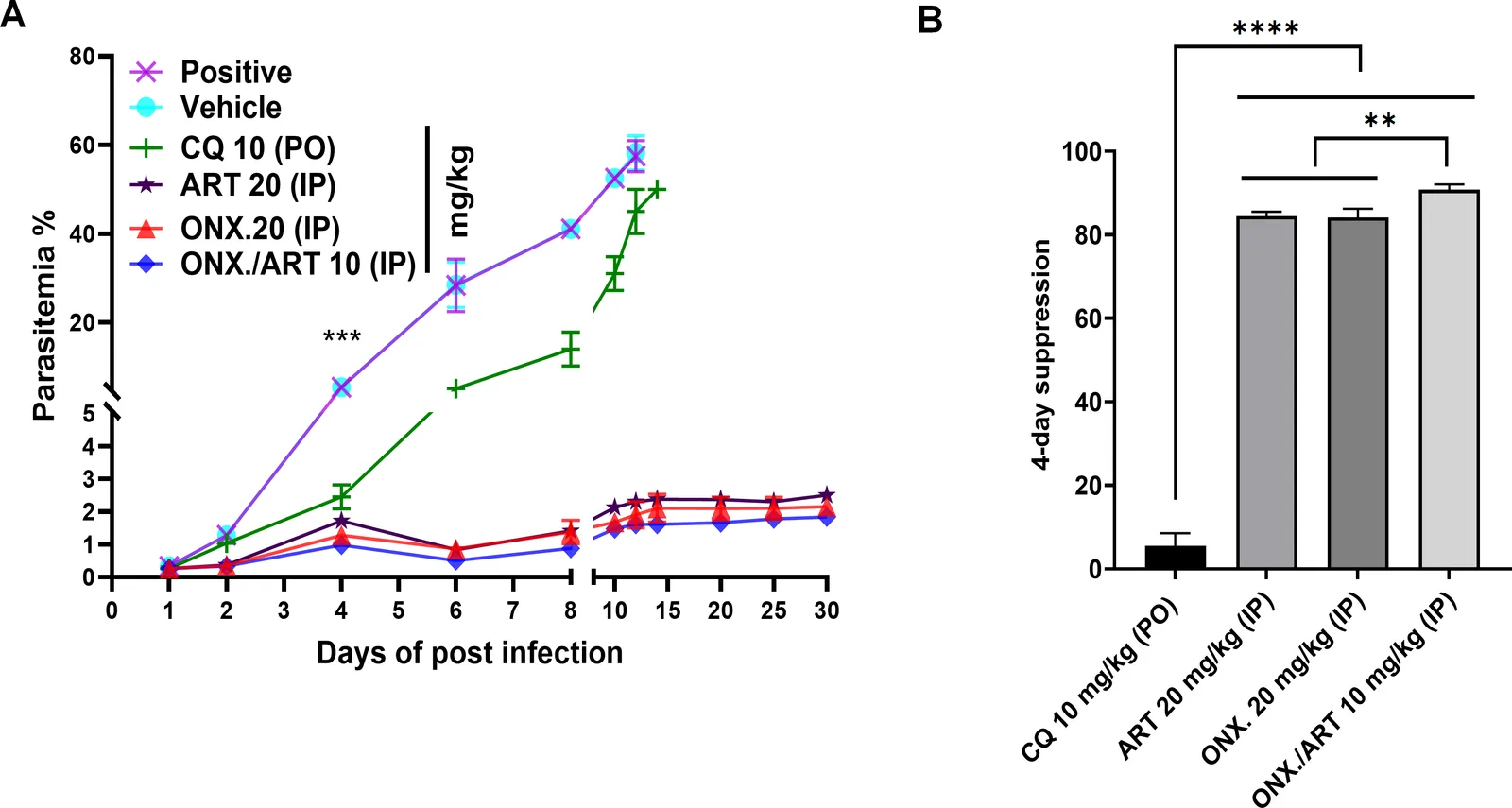
Parasitemia and suppression. (**A**) Parasitemia kinetics were monitored up to 32 DPI. Parasitemia was determined by microscopy of at least 1,000 red blood cells. (**B**) Parasitemia suppression after 4 days of treatment was calculated using the following equation: (% 4-day suppression = 100 − 100 × (parasitemia of intervention/parasitemia positive). Intervention (infected mice with treatment) and positive (infected mice without treatment). ONX-0914 significantly increased 4-day suppression when administered as monotherapy or in combination therapy with ART compared to CQ. The ONX-0914/ART combination outperformed monotherapy. Statistical significance was calculated using a two-tailed *t*-test (*P < 0.01, **P < 0.001, ***P < 0.0001, and ****P < 0.00001). The data are shown as the mean ± SD. Each group included five mice.

The parasitemia of *P. yoelii* decreased rapidly after 4 days of treatment with the ONX/ART combination, reaching levels below 0.6%. Subsequently, both ONX-0914 and ART monotherapies kept parasitemia levels below 1.0% (Fig. 5A and B), consistent with their rapid *in vitro* activity. Similarly, parasitemia remained below 1.9% up to 30 days post-treatment in combination therapy, followed by ONX and ART monotherapy groups (<2.5%), indicating sustained antiplasmodial efficacy (Fig. 5A). By 6 DPI, ONX-0914/ART combination suppressed parasitemia by nearly 90%, yielding significantly greater inhibition than ONX and ART monotherapy (⁓84%, *P* < 0.05; Fig. 5B). These findings support the efficacy of ONX-0914 in *vivo* and enhance ACT effect via IP route against CQ-resistant strain parasites.

**Fig 6:**
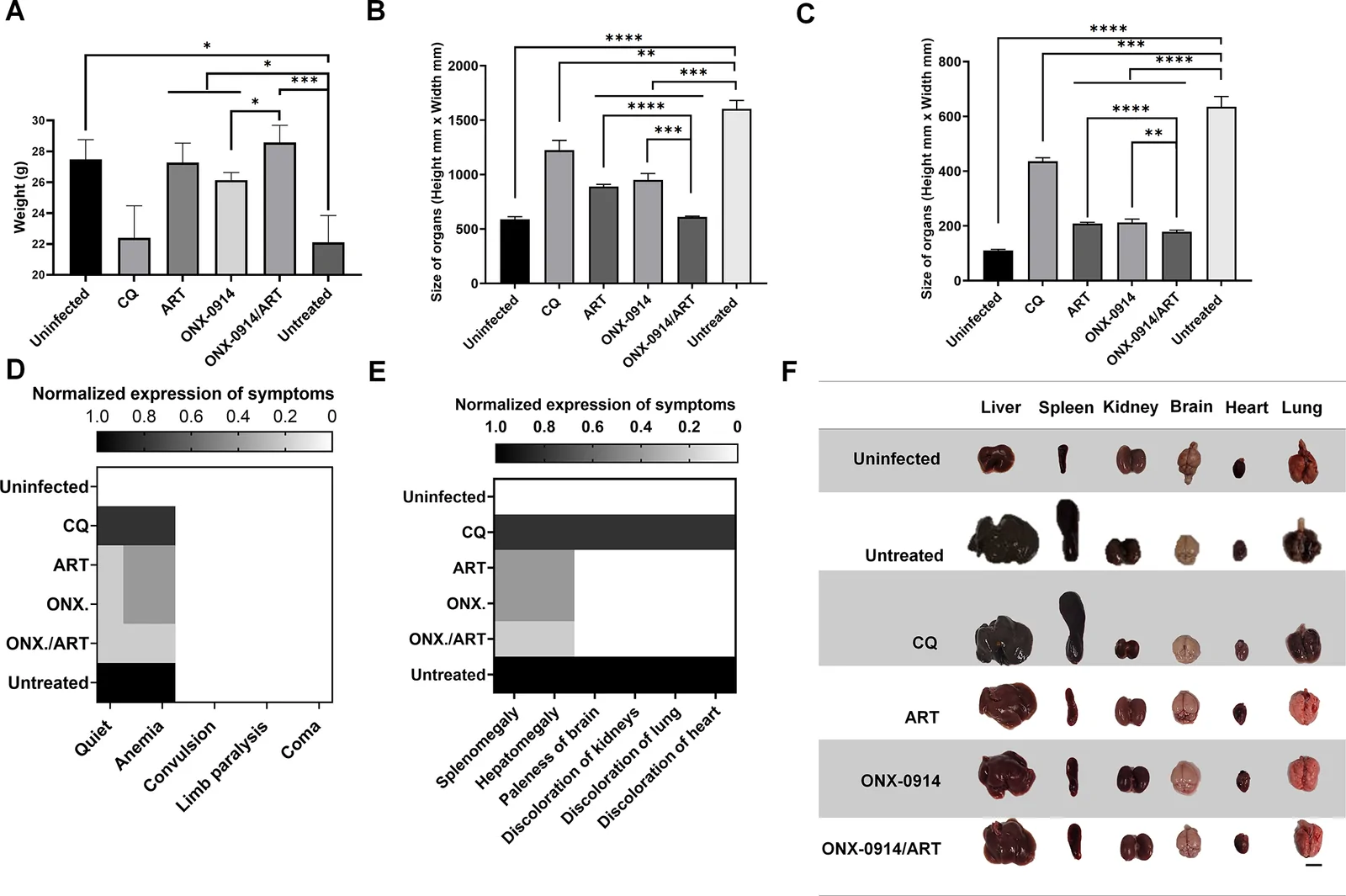
Clinical parameters and organ pathology. (**A**) Weights of the mice at 15 DPI. ONX-0914 monotherapy and the ONX-0914/ART combination both improved body weight compared with untreated mice. (**B and C**) Liver and spleen sizes. Organ size was calculated as height × width (mm). Mice treated with ONX-0914 alone or in combination with ART presented smaller livers and spleens compared with untreated mice. (**D**) Clinical symptoms. Symptom prevalence was expressed as the percentage of mice with symptoms relative to the total number of mice in each group. (**E**) Gross pathological features of organs (brain, heart, lung, liver, kidneys, and spleen) after sacrifice, after 15 days (color and size). (**F**) Images of the brain, heart, lung, liver, kidney, and spleen. Scale bar = 5 mm. Negative (uninfected mice) and positive (infected mice). Organs were collected from three mice per group. (**A–C**) Statistical significance calculated using a two-tailed *t*-test is expressed relative to the positive and vehicle (infected mice and infected mice treated with vehicle) using asterisks (*P < 0.01, **P < 0.001,***P < 0.0001 and ****P < 0.00001).

As a result of reduced parasite burden, treatment with ONX-0914 monotherapy or the ONX/ART combination improved body weight, clinical signs, and organ function. Weight loss was significantly attenuated in mice receiving ONX-0914, ART, or ONX/ART combination by 14 DPI (*P* < 0.05; Fig. 6A). Additionally, mice treated with the ONX/ART combination exhibited fewer clinical symptoms—including inactivity, ruffled fur, and anemia, compared with ONX-0914 monotherapy (Fig. 6D). Hepatomegaly and splenomegaly, which were common in the control group, were markedly reduced in both the ONX-0914 and ONX/ART treatment groups (Fig. 6E). Treated mice displayed smaller livers and spleens without tissue discoloration, indicating protection against *Plasmodium*-associated organ pathology (Fig. 6B, C and F; *P* < 0.001). Histopathological lesions caused by *P. yoelii* were also substantially less severe in mice treated with ONX-0914 or the ONX/ART combination (Table 2; Fig. S1). Vascular congestion and hemorrhage in the brain, heart, lungs, liver, spleen, and kidneys—frequently observed in control mice—were absent in treated mice. Livers from infected controls showed diffuse pigmentation and increased hematopoietic precursor cells, features absent in the ONX/ART-treated mice, further supporting the protective effect of combination therapy.

**TABLE 2:** Histological findings in various organs from experimental parasite-infected mice^*a*^^,*b*^

Organ and histopathology/test materials	Positive	CQ 10 mg/kg PO	ART 20 mg/kg IP	ONX 20 mg/kg IP	ONX/ART 10 mg/kg IP
Brain			NSL	NSL	NSL
Stasis in capillaries	P	P			
Bleeding	P	P			
Pigments in blood (hemozoin deposits)	3+	3+			
Precursor cells in blood	2+	2+			
Heart					NSL
Pigments in blood (hemozoin deposits)	3+	3+			
Precursor cells in blood vessel	2+	2+			
Pigments in blood vessel			1+	1+	
Lung					NSL
Pigments in alveolar walls	3+	3+			
Pigments in blood (hemozoin deposits)	4+	4+			
Precursor cells in blood	2+	2+			
Hemorrhage and pigments, focal			1+	1+	
Liver			NSL	NSL	NSL
Pigments, Kupffer cells	4+	4+			
Pigments, blood (hemozoin deposits)	3+	4+			
Precursor cells in blood	3+	3+			
Hepatocytic vacuolation	3+	3+			
Spleen					
Pigments, red pulps	3+	3+	1+	1+	
Extramedullary hematopoiesis	4+	4+			
Apoptotic cells, white pulp	2+	3+	1+	1+	1+
Kidney			NSL	NSL	NSL
Pigments in blood (hemozoin deposits)	3+	3+			

^*a*^ NSL, no specific lesion.

^*b*^ Criteria for evaluation: 1+, minimal; 2+, mild; 3+, moderate; 4+, severe.

## DISCUSSION

The growing emergence of resistance to frontline antimalarial drugs underscores the urgent need to identify therapeutics with novel mechanisms of action. The MMV has focused on developing compounds with potent antiplasmodial activity against various *Plasmodium* spp., efficacy against resistant strains, and synergistic interactions with existing antimalarial agents (26, 42). Recent antimalarial drug-discovery efforts have yielded several mechanistically distinct inhibitors and dihydroorotate dehydrogenase inhibitors. Nevertheless, the continued expansion of antimalarial drug discovery remains essential to address emerging resistance and support future combination therapy (42–45). In this context, UPS has been identified as a target because of its essential role in parasite protein homeostasis and survival (16–18). PIs demonstrated potent antimalarial activity against multiple *Plasmodium* species, including the ART-resistant *P. falciparum* laboratory strain evaluated in this study and the CQ-resistant *P. yoelii* model evaluated, and synergized with ART and other proteasome inhibitors, supporting further investigation of PIs as antiplasmodial lead compounds.

Proteasome inhibitors exhibited cytotoxic effects in mammalian systems, raising concerns regarding host toxicity and the need for compounds with enhanced selectivity toward the target proteasome. ONX-0914 has been reported to retain potent proteasome inhibition while demonstrating improved tolerability in preclinical studies (46, 47). Similarly, epoxomicin exhibited highly selective binding to the 20S proteasome, as demonstrated by crystallographic analyses, and demonstrated potent antiplasmodial activity (48, 49). LU-005i also displayed promising immunoproteasome-selective inhibition with low cytotoxicity (50). Bortezomib is approved for hematologic malignancies; however, its clinical use is limited by dose-dependent adverse effects, including peripheral neuropathy (51). Our cytotoxicity analysis in HEK293T cells identified epoxomicin, ONX-0914, and LU-005i as high-selectivity candidates (CC_50_ > 100 µM, SI > 5,000). However, these findings should be interpreted as preliminary evidence of *in vitro* selectivity rather than comprehensive evidence of safety. Additional studies in diverse mammalian cell types and animal models, together with further structure optimization, will be necessary to define safety profiles, therapeutic windows, and translational potential for malaria treatment.

The next generation of antimalarial drugs is expected to exhibit broad efficacy against diverse parasite strains in accordance with the MMV target (26). Drug susceptibility varies not only among *Plasmodium* species but also among strains within the same species (52). For example, *P. knowlesi* is more susceptible than *P. falciparum* to dihydrofolate reductase inhibitors such as pyrimethamine, cycloguanil, and trimethoprim, while showing reduced sensitivity to agents including blasticidin and DSM1 (53). UPS is a conserved and essential pathway regulating protein homeostasis across *Plasmodium* species (13–15). PIs have therefore emerged as promising antiplasmodial agents with activity against the blood-stage parasites. Specifically, lactacystin and its analog show potent activity against *P. falciparum*, *P. berghei*, and *P. yoelii* (54). Consistent with these observations, our study revealed significant inhibition of epoxomicin, ONX-0914, and LU-005i against the Pf20S and Pk20S proteasomes (IC_50_ < 50 nM), which correlated with strong antiplasmodial activity against both species (IC_50_ < 100 nM). Although *P. knowlesi* was more sensitive to ONX-0914 and LU-005i than *P. falciparum*, the consistent activity observed across *P. falciparum*, *P. knowlesi*, and the *P. yoelii* model supports the concept that disruption of parasite protein homeostasis is a broadly applicable antiplasmodial strategy. The comparative approach adopted in this study was based on the high conservation of the UPS across *Plasmodium* species rather than the expectation of identical drug susceptibility. Importantly, because robust continuous *in vitro* culture of *P. vivax* remains unavailable, evaluation in complementary *Plasmodium* species with conserved UPS biology provides a practical framework for assessing whether proteasome-targeting compounds may demonstrate broader efficacy against human malaria parasites. However, the extent to which such activity is maintained across genetically diverse clinical isolates and drug-resistant parasite populations remains to be determined.

Mechanistically, the observed antimalarial activity was associated with inhibition of proteasome activity and disruption of the ubiquitin-proteasome system. Although these findings support proteasome inhibition as a likely mechanism, direct target engagement was not demonstrated. Further studies employing chemical proteomics, target-occupancy approaches, structural analyses, or resistance-selection experiments will be crucial for confirming target engagement and determining the molecular basis of antiplasmodial activity. Previous studies have shown that PIs such as SW584, MG132, and epoxomicin exhibit rapid parasite clearance and disrupt multiple stages of the parasite life cycle (21, 55). Our study confirmed that epoxomicin, ONX-0914, and LU-005i effectively inhibited parasite growth across multiple stages of both *P. falciparum* and *P. knowlesi*. Epoxomicin and ONX-0914 induced early pyknotic changes in ring-stage *P. knowlesi* parasites within 6–12 h, suggesting rapid activity. Quantitative kill-rate measurements were assessed against PfNF54luc-GFP, as a luciferase-expressing PkA1H1 line was not available, and demonstrated potent inhibition within 6 h. The lack of direct kinetic measurements in PkA1H1 represents a limitation of this study. In accordance with established criteria, compounds are classified as fast-acting when they block the parasite at early stages, whereas slow-acting inhibitors allow the parasite to reach late stages (40). Collectively, the rapid clearance kinetics and multi-stage activity observed in the parasite models examined support further evaluation of PIs as antimalarial agents targeting the asexual blood stages. The observed effects are consistent with disruption of the *Plasmodium* ubiquitin-proteasome system, although direct binding has not been confirmed. Although multiple *Plasmodium* species were investigated, the present study was restricted to asexual blood-stage models. Therefore, no conclusions can be drawn regarding activity against liver stages, sexual stages, transmission stages, or relapse-associated species such as *P. vivax* and *P. ovale*. Further studies should assess the activity of PIs across additional parasite developmental stages and clinically relevant *Plasmodium* species to better identify their antimalarial spectrum.

Combination therapy remains central to malaria treatment, and synergistic drug interactions may improve efficacy, decrease drug exposure, and potentially delay the emergence of resistance. *In vitro* studies have demonstrated strong synergistic interactions between ART and several proteasome inhibitors, including TDI-8304, epoxomicin, and SW584, against *P. falciparum* (24, 38). *In vivo*, TDI4258 significantly reduced parasitemia when combined with WLW-VS in a *P. yoelii* mouse model, compared with monotherapy (23). In the present research, the ART-resistant PfDd2R539T(+) strain was primarily included to assess the interaction between ONX-0914 and ART in a resistant genetic background rather than to evaluate susceptibility to artemisinin resistance itself. We demonstrated potent synergistic interactions between ONX-0914 and ART, as well as LU-005i, against ART-resistant PfDd2R539T(+) and *P. knowlesi in vitro* ( ∑FIC50< 0.8), supporting further evaluation of proteasome inhibitor-based combination regimens across diverse parasites. However, ART resistance was not determined using the ring-stage survival assay, which remains the accepted method for evaluating the clinically relevant phenotype of partial artemisinin resistance. Therefore, these findings should not be interpreted as evidence of efficacy against partially ART-resistant parasites, and future studies incorporating RSA and additional clinical isolates carrying validated K13 mutations will be essential.

In contrast, ONX-0914 and epoxomicin demonstrated antagonism in PfDd2R539T(+) but synergy in PkA1H1. This strain-specificity may reflect subunit selectivity and altered proteasome composition in the resistant strain. Epoxomicin primarily targets the chymotrypsin-like β5 subunit of the 20S proteasome, whereas ONX-0914 preferentially inhibits the immunoproteasome-associated β5i subunit but can also affect β5 activity under certain conditions. Competitive binding within a structurally altered β5 active site in the resistant strain may reduce cooperative inhibition and manifest antagonism, whereas preserved subunit architecture in PkA1H1 may permit complementary inhibition and synergy. Although not directly validated here, these findings suggest that proteasome subunit composition and mutation status may influence combinatorial responses.

Importantly, ONX-0914 enhanced the efficacy of the ACT regimen against CQ-resistant *P. yoelii in vivo*, resulting in sustained parasite suppression and improved clinical outcomes, underscoring the potential of PI-based combination therapies to improve antimalarial efficacy. Nevertheless, these *in vivo* findings should be interpreted as proof of concept. A limitation in this study is that *in vivo* efficacy was determined exclusively using intraperitoneal administration in the *P. yoelii* model. This route was administered to minimize variability in systemic drug exposure and to facilitate assessment of the pharmacodynamic interaction between ONX-0914 and ART, which exhibits poor oral bioavailability in rodents. However, IP does not reflect the clinically relevant route of antimalarial administration, thereby limiting the direct translation of the present findings. Furthermore, pharmacokinetic parameters and systemic drug exposure were not evaluated, precluding assessment of exposure-response relationships. Consequently, the *in vivo* findings should be interpreted as proof of concept for efficacy rather than as a quantitative assessment of pharmacodynamic potency. Evaluation of oral efficacy and pharmacokinetic properties will be essential for determining the translational potential of these compounds. Further studies investigating the combination of ONX-0914 with additional PIs, comprehensively assessing activity against a broader panel of genetically diverse and drug-resistant *Plasmodium* isolates, and exploring resistance mechanisms, including *Plasmodium* 20S mutations, may enhance therapeutic development.

## SUPPLEMENTAL MATERIAL

Fig. S1 aac.00382-26-s0001.docx Histopathological examination.

ASM does not own the copyrights to Supplemental Material that may be linked to, or accessed through, an article. The authors have granted ASM a non-exclusive, world-wide license to publish the Supplemental Material files. Please contact the corresponding author directly for reuse.
