# Supplementary material for: Anti-plasmodial activity of proteasome inhibitors against various Plasmodium species: a preclinical evaluation
Source: Antimicrob Agents Chemother. 2026 Sep 30;70(11):e00382-26. doi: 10.1128/aac.00382-26 (PMC13731232; doi:10.1128/aac.00382-26)
Supplement: Supplementary file 1 [file aac.00382-26-s0001.docx]

**SUPPLEMENTARY FIG 1** Histopathological examination of *P. yoelii*-infected mice treated with antimalarial drugs. (A) Histological analysis of the liver. (B) Histological analysis of the spleen. (C) Histological analysis of the kidney. (D) Histological analysis of the brain. (E) Histological analysis of the heart. (F) Histological analysis of the lung. The common signs of lesions were brown‒yellowish pigmentation (thin arrows), which was evident in macrophages and in blood vessels, and bars=50 µm (A-F). Other pigmentation (thick arrows) was observed in alveoli, thrombi (F), sinusoids, and haematopoietic aggregates (A**)**, and haematopoietic precursors in red pulp (B**)**. Lesions were not observed in the negative, ONX/ART combination groups. Negative (uninfected mice), positive (infected mice). H&E-stained images.
